# The Way I Paint—How Image Composition Emerges During the Creation of Abstract Artworks

**DOI:** 10.1177/2041669520925099

**Published:** 2020-05-09

**Authors:** Christoph Redies

**Affiliations:** Experimental Aesthetics Group, Institute of Anatomy I, University of Jena School of Medicine

**Keywords:** experimental aesthetics, painting, creative process, image properties, low-level visual processing, deep neural network, abstract art.

## Abstract

In recent years, there has been an increasing number of studies on objective image properties in visual artworks. Little is known, however, about how these image properties emerge while artists create their artworks. In order to study this matter, I produced five colored abstract artworks by myself and recorded state images at all stages of their creation. For each image, I then calculated low-level features from deep neural networks, which served as a model of responses properties in visual cortex. Two-dimensional plots of variances that were derived from these features showed that the drawings differ greatly at early stages of their creation, but then follow a narrow common path to terminate at or close to a position where traditional paintings cluster in the plots. Whether other artists use similar perceptive strategies while they create artworks remains to be studied.

Much is known about the role of the art historical context, aesthetic concepts, artistic intentions, and other cultural factors on creating artworks. However, the formal structure (or composition) of artworks can also play a role in mediating aesthetic perception (Redies, 2015). How this composition is affected by perceptual strategies and decisions taken by artists during the creation of visual artworks is largely unknown.

Most traditional artworks are produced during a generative process, by which an artist composes an artwork in a step-by-step fashion, until she or he is finally satisfied with the creation. Of course, there are numerous variations and exceptions from this general scheme, particularly in modern art (e.g., *readymades* or *objects trouvés*).

One way to gain insight into the creative process is to analyze complete series of state images that are recorded throughout the creative process. One of the first examples for this type of approach was a study on four state images of a drip painting by Jackson Pollock (Taylor et al., 2002; for a more comprehensive study, see Redies et al., 2015). In order to produce state series, photographs or scans need to be taken under reproducible and consistent conditions. Unfortunately, such high-quality datasets are rare in the art world. In view of this shortage, I resorted to digitize state proofs of artworks that I produced by myself. This approach is problematic because of my dual roles as creator of the drawings and experimenter, which should be independent.

For the present study, I produced five abstract drawings with color pencils and watercolors. My goal was to achieve a well-balanced and harmonious composition of color, lines, edges, patches, circles, and so on. I finished the drawings when I felt that an aesthetically pleasing composition had been reached, based on purely subjective (nonexplicit) grounds. Being abstract, the drawings do not have any figurative or conceptual meaning. They are here called Drawing 1 to Drawing 5 and were produced largely in parallel during multiple short time periods between April 2014 and May 2019. No objective measurements were carried out on any of the drawings until all drawings had been completed (or almost completed).

During the creation of each drawing, I obtained complete series of digitized state images with a calibrated scanning device (Perfection V700 Photo, Epson; range, 43-79 state images). Exemplary state images and the final version of three of the drawings are depicted in Figure 1. The accompanying online video^1^ shows complete series of consecutive state images for each of the five drawings.

**Figure 1.**
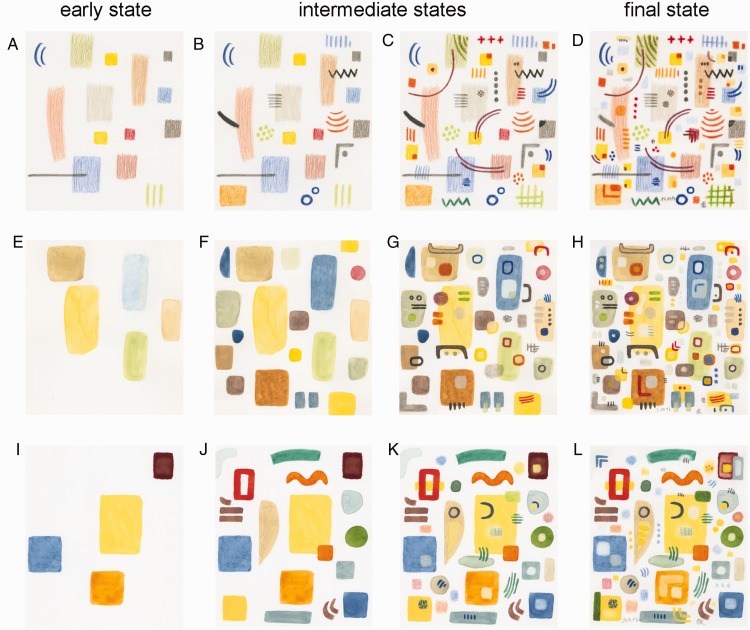
Examples of the abstract drawings (D, Drawing 1; H, Drawing 4; and, L, Drawing 5) and their early and intermediate state versions (A–C, E–G, and I–K). The position of these images in the variance plot is shown in Figure 2 (open circles).

To quantify image structure, I calculated responses of low-level convolutional neural network filters. The filter responses resemble visual cortical responses to oriented luminance changes, color patches, and spatial frequency content (for a more detailed description of the method, see Brachmann et al., 2017). We have shown previously that traditional artworks are characterized by high Richness and intermediate levels of Variability of the filter responses across an image (Brachmann et al., 2017). Figure 2 shows a plot of the two measures. Richness is defined as the opposite of the total variance of all convolutional neural network filter responses across all segments of an image (*x*-axis of the plot). The smaller this measure, the more pictorial elements are distributed all over the image, that is, image structure is richer. The *y*-axis indicates the median over the variances of each filter across all segments of the image (here called Variability). Higher values indicate that the pictorial elements are less self-similar across the image, that is, the image structure is more variable.

**Figure 2.**
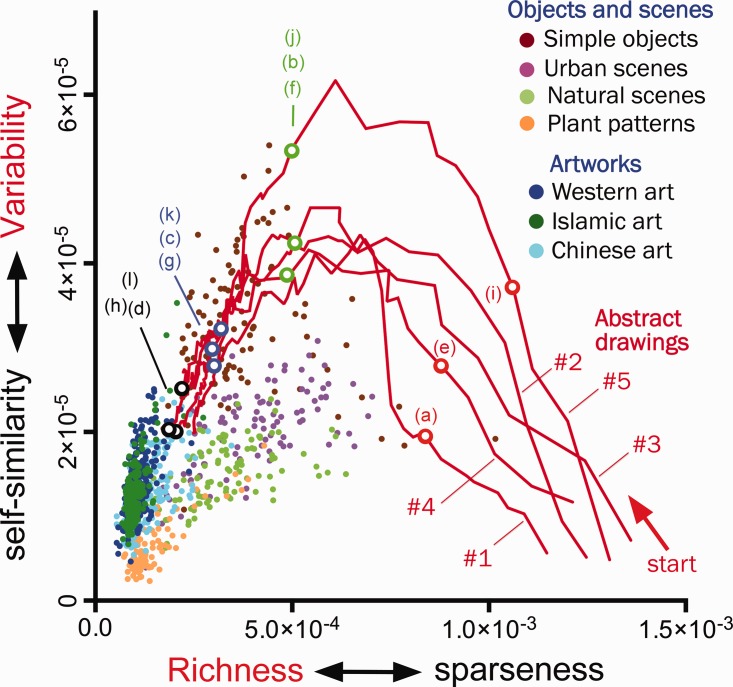
Summary plot of Richness and Variability (Brachmann et al., 2017) for all state versions of the five abstract drawings (red lines). As shown step-by-step in the online video,^1^ the red lines trace how the variances change during the production of the drawings, beginning at the lower right corner of the plots and finalizing at or close to the cluster of the artworks at the left-hand side. The open dots indicate the state images shown in Figure 1 (*red*, early states; *green* and *blue*, intermediate states; and *black*, final states), as indicated. As a reference, the differently colored closed dots show results for photographs of objects, scenes, and artworks, respectively. Each dot represents one image. Note that if two images assume a similar position in this plot, they do not necessarily look the same because other image properties also contribute to the visual appearance of the images.

Note that traditional artworks of Western, Islamic, and Chinese provenance form a distinct cluster that is largely separate from the other types of images. The red lines in Figure 2 indicate how each drawing emerges stepwise, starting with the first pictorial element on the lower right-hand side of the plot and terminating with the final state of the drawing on the left-hand side. The online video^1^ shows how the curves develop in parallel to the state images.

Unsurprisingly, Richness increases steadily as more and more pictorial elements are added during the drawing process. Variability increases initially in all five drawings, but to different degrees. All plots then change direction and drop to low or intermediate values of Variability. The plot positions of the five drawings show considerable diversity at early stages. This finding correlates with my subjective impression that the freedom for composing pictorial elements was large at the beginning of the creative process, but narrowed down drastically towards the end of the process. Correspondingly, as image composition grows richer, the curves of all drawings become more uniform and follow a narrow common path that terminates at or close to the cluster of the traditional artworks.

In conclusion, the present results demonstrate that an artist’s creative decisions can be studied by analyzing objective image properties that relate to visual processing in the human brain. However, the present study is restricted to two image properties and one particular style of abstract art. To what extent other artists use similar perceptive strategies while they create artworks remains to be studied. Undoubtedly, the same properties will be less useful for analyzing some other styles of abstract art, such as color field paintings, where pictorial elements are neither rich nor variable. Last but not least, future experiments need to separate the roles of creator and experimenter more clearly, for example, by having naïve persons compose artworks from a standardized set of pictorial elements (Letsch and Hayn-Leichsenring, 2018).

## Note

1 An online video, which explains the variance plot and shows the full sequence of state images and the corresponding plots, can be accessed at the Figshare repository: https://doi.org/10.6084/m9.figshare.12130455
